# Feedlot diets containing different starch levels and additives change the cecal proteome involved in cattle’s energy metabolism and inflammatory response

**DOI:** 10.1038/s41598-022-09715-7

**Published:** 2022-04-05

**Authors:** Leone Campos Rocha, Andrey Sávio de Almeida Assunção, Renata Aparecida Martins, Victor Valério de Carvalho, Alexandre Perdigão, Marília Afonso Rabelo Buzalaf, Jiri Adamec, Camila Pereira Braga, Danilo Domingues Millen, José Cavalcante Souza Vieira, Pedro de Magalhães Padilha

**Affiliations:** 1 School of Veterinary Medicine and Animal Science, São Paulo State University (UNESP), Botucatu, São Paulo Brazil; 2 DSM Nutritional Products SA, São Paulo, Brazil; 3 Bauru School of Dentistry, University of São Paulo (USP), Bauru, São Paulo, Brazil; 4 Department of Biochemistry, University of Nebraska-Lincoln (UNL), Lincoln, USA; 5 College of Technology and Agricultural Sciences, UNESP, São Paulo State University, Dracena, São Paulo, Brazil; 6 Institute of Biosciences, São Paulo State University (UNESP), Botucatu, São Paulo Brazil; 7 Laboratory of Bioanalytical and Metalloproteomic, Department of Chemistry and Biochemistry, Institute of Biosciences, São Paulo State University, Botucatu, São Paulo 18618-693 Brazil

**Keywords:** Proteomics, Epigenomics

## Abstract

Diets for feedlot cattle must be a higher energy density, entailing high fermentable carbohydrate content. Feed additives are needed to reduce possible metabolic disorders. This study aimed to analyze the post-rumen effects of different levels of starch (25%, 35%, and 45%) and additives (monensin or a blend of essential oils and exogenous α-amylase) in diets for Nellore feedlot cattle. The cecum tissue proteome was analyzed via two-dimensional polyacrylamide gel electrophoresis (2D-PAGE) and then differentially expressed protein *spots* were identified with liquid chromatography–tandem mass spectrometry (LC–MS/MS). The use of blends of essential oils associated with α-amylase as a feed additive promoted the upregulation of enzymes such as triosephosphate isomerase, phosphoglycerate mutase, alpha-enolase, beta-enolase, fructose-bisphosphate aldolase, pyruvate kinase, glyceraldehyde-3-phosphate dehydrogenase (GAPDH), l-lactate dehydrogenase B, l-lactate dehydrogenase A chain, l-lactate dehydrogenase, and ATP synthase subunit beta, which promote the degradation of carbohydrates in the glycolysis and gluconeogenesis pathways and oxidative phosphorylation, support pyruvate metabolism through the synthesis of lactate from pyruvate, and participate in the electron transport chain, producing ATP from ADP in the presence of a proton gradient across the membrane. The absence of proteins related to inflammation processes (leukocyte elastase inhibitors) in the cecum tissues of animals fed essential oils and amylase may be because feed enzymes can remain active in the intestine and aid in the digestion of nutrients that escape rumen fermentation; conversely, the effect of monensin is more evident in the rumen and less than 10% results in post-ruminal action, corroborating the hypothesis that ionophore antibiotics have a limited effect on the microbiota and intestinal fermentation of ruminants. However, the increase in starch in these diets promoted a downregulation of enzymes linked to carbohydrate degradation, probably caused by damage to the cecum epithelium due to increased responses linked to inflammatory injuries.

## Introduction

Among the limitations to enhancing meat production is its large energy requirement, which means that feedlot cattle have a higher net energy demand (NE)^1^. Dietary strategies are adopted to increase energy metabolism, mainly through the fermentation of carbohydrates in the rumen. This is mostly accomplished by supplying hexoses (glucose) from starch. Thus, with increased starch in the diet, physiological limits to animals’ ability to digest a large amount of fermentable carbohydrates in the rumen and ruminal escape increased.

In the rumen, the fermentation of glucose from starch occurs and it is converted mainly into volatile fatty acids (AGV) and lactate^2^, which are metabolized in the liver and provide the greatest source of energy for ruminants^3,4^. However, the use of large amounts of starch can lead to disorders and metabolic diseases due to the accumulation of organic acids in the ruminal fluid, especially acidosis and bloat (NASCEM^5^). Thus, feed additives that decrease harmful ruminal fermentation processes are employed, such as sodium monensin, which is a polyester carboxylic ionophore used in growth and finishing diets^5^. It acts bacteriostatically on Gram-positive ruminal bacteria but may leave residues in products of animal origin and result in microbial resistance^6^. Alternative additives have shown the potential to replace monensin, such as blends of essential oils associated with the exogenous enzyme α-amylase, which has led to demonstrable gains in performance and carcass weight, in addition to reducing hepatic abscesses and fecal starch in animals with high-starch diets^7–9^.

With high levels of starch in the diet, the rate of passage and post-ruminal digestion increases^5^. The rumen microbiota can digest around 70–80% of the starch consumed^5,10–13^; however, the digestion and absorption of post-rumen starch are partially impaired as enzymatic digestion via pancreatic α-amylase in the duodenum is limited to the small intestine^14,15^. Others have postulated that glucose cannot be absorbed and transported in large quantities from the lumen into the bloodstream due to insufficient levels of the glucose transporters SLGT1 and GLUT2^5,16,17^, which favors the escape of some starch to the large intestine and increases the potential for the digestion and use of this starch in the cecum. Therefore, feedlot diets that offer increased amounts of energy due to high levels of concentrates^18^ can cause excessive fermentation in the cecum, contributing to the metabolizable energy ruminants can access^19,20^ but potentially resulting in hindgut acidosis, which may generate inflammatory reactions in the cecal epithelium. Large amounts of starch in the cecum may contribute to the fermentation of AGV, NH_3_, and lactic acid as well as a decreased pH^5^. Additionally, the cecum has limited buffering capacity compared with the rumen, where saliva and protozoa modulate pH fluctuations^21^. Feed additives that can increase the use of starch in the rumen, reducing starch escape to the intestines, as well as lower starch levels in feedlot cattle diets can avert the risk of cecal acidification. Most studies with feed additives focus only on the rumen effects; however, the effects of feed additives on the post-rumen digestive tract are important to understand.

Understanding how the digestion and absorption sites act when high proportions of starch are included in feedlot diets is essential. Due to the levels of starch in cattle diets and the effects on the extent of the gastrointestinal tract associated with different feed additives, this study aims to map the proteome of feedlot cattle’s ceca and to elucidate how protein expression acts on metabolism when different nutritional strategies are applied.

## Results

### Image analysis and protein expression

In the workspace, classes were created to analyze differences in protein expression; an analysis of variance (ANOVA) was used to test the hypothesis (H_θ_) that the expressed *spots* are identical. When testing all classes, protein *spots* were differentially expressed, as shown in Table 1.

**Table 1:** Differentially expressed *spots* in Nellore beef cattle cecum fed with diets containing increasing starch levels (25, 35, and 45%) and additives (Monensin, Blend of essential oil + exogenous α-amylase).

SPOT (n)	MON × BEO*			MON*			BEO*		
	25 × 25	35 × 35	45 × 45	25 × 35	35 × 45	25 × 45	25 × 35	35 × 45	25 × 45
Up	9	3	7	14	3	8	5	0	1
Down	11	16	5	6	28	4	10	6	13
+	10	59	14	22	65	35	34	16	27
∅	37	11	14	81	19	42	18	8	16
Total	67	89	40	125	115	89	67	30	57

*UP* up-regulated *spot*, *Down* down-regulated *spot*, + *spot* present in the first group in relation to the second, ∅ *spot* absent in the first group in relation to the second.

*$$P \le 0.05$$.

Supplemental Figure S2 shows the distribution of proteins and their biological processes, molecular functions, and cellular components.

Proteins associated with glucose metabolism and energy synthesis (Table 2) and macromolecules involved in the degradation of carbohydrates through the glycolytic pathway, gluconeogenesis, and oxidative phosphorylation were detected in cecal tissue. The expression of seven enzymes participating in the glycolysis and gluconeogenesis pathways was verified: triosephosphate isomerase (Step 1); phosphoglycerate mutase (Step 2); alpha-enolase (ENO1), beta-enolase (ENO3), and fructose-bisphosphate aldolase (ALDOB) (Step 4); pyruvate kinase (PKM) (Step 5); and glyceraldehyde-3-phosphate dehydrogenase (GAPDH). Three enzymes linked to pyruvate metabolism or catalytic activities participating in the synthesis of lactate from pyruvate were verified as well: l-lactate dehydrogenase B, l-lactate dehydrogenase A chain, and l-lactate dehydrogenase. ATP synthase subunit beta participated in the electron transport chain, producing ATP from ADP in the presence of a proton gradient across the membrane.

**Table 2:** Proteins identified by LC–MS/MS in protein spots differentially expressed in Nellore bovine cecum fed on diets containing increasing levels of starch (25, 35 and 45%) and additives (monensin, blend essential oil + exogenous α-amylase).

Protein	Access	Score	pI/MM theoretical (Da)	pI/MM experimental (Da)
**Glucose and energy metabolism**				
Alpha-enolase	Q9XSJ4	1783.3310	6.37/47,326.13	6.70/56,906
Beta-enolase	Q3ZC09	440.2993	7.60/47,096.01	6.43/48,539
Triosephosphate isomerase	Q5E956	193.3130	6.45/26,689.51	7.24/25,458
l-lactate dehydrogenase B	Q5E9B1	4599.0320	6.02/36,723.64	6.37/39,211
l-lactate dehydrogenase A chain	P19858	1327.3960	8.12/36,597.64	6.37/39,211
Pyruvate kinase	A5D984	98.4805	7.96/57,948.91	5.9/57,613
Fructose-bisphosphate aldolase	A6QLL8	1850.8330	8.45/39,436.12	6.37/39,211
Phosphoglycerate mutase	F1N2F2	427.2343	9.01/28,699.04	6.37/39,211
l-lactate dehydrogenase	F1MK19	70.7983	5.72/36,724.58	6.37/39,211
Glyceraldehyde-3-phosphate dehydrogenase	P10096	11,907.1000	8.51/35,868.09	8.12/29,321
ATP synthase subunit beta_ mitochondrial	P00829	533.0471	5.15/56,283.53	5.49/47,920
**Inflammatory response**				
Leukocyte elastase inhibitor	Q1JPB0	300.0084	5.70/42,235.75	5.70/38,338

### Pathways enrichment and Reactome analysis

**Figure 1:**
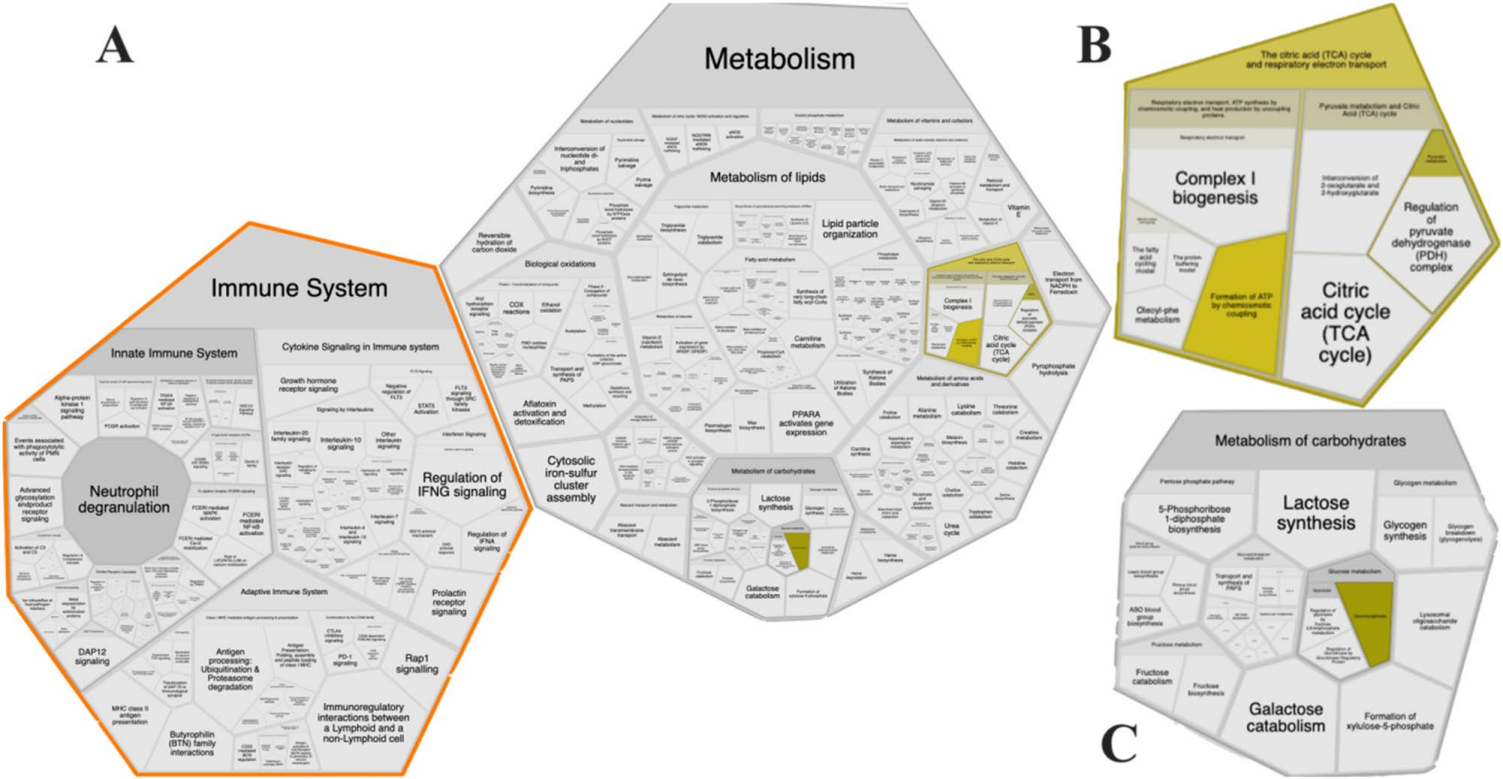
Affected pathways generated from KEGG ID input using Reactome show that immune system and metabolism is impacted (**A**), which, glycolysis and gluconeogenesis (**B**) and metabolism of carbohydrates (**C**).

The pathway enrichment and Reactome analysis yielded similar results showing that specific pathways were affected. The differential expression found in all groups displayed changes in metabolic pathways as carbohydrate metabolism, pyruvate metabolism, the citric acid (TCA) cycle, respiratory electron transport, innate immune system, and the immune system were affected in cecum tissues by different feeding strategies (Fig. 1). The data from the Reactome pathway analysis have been provided in Supplemental Table S1.

**Figure 2:**
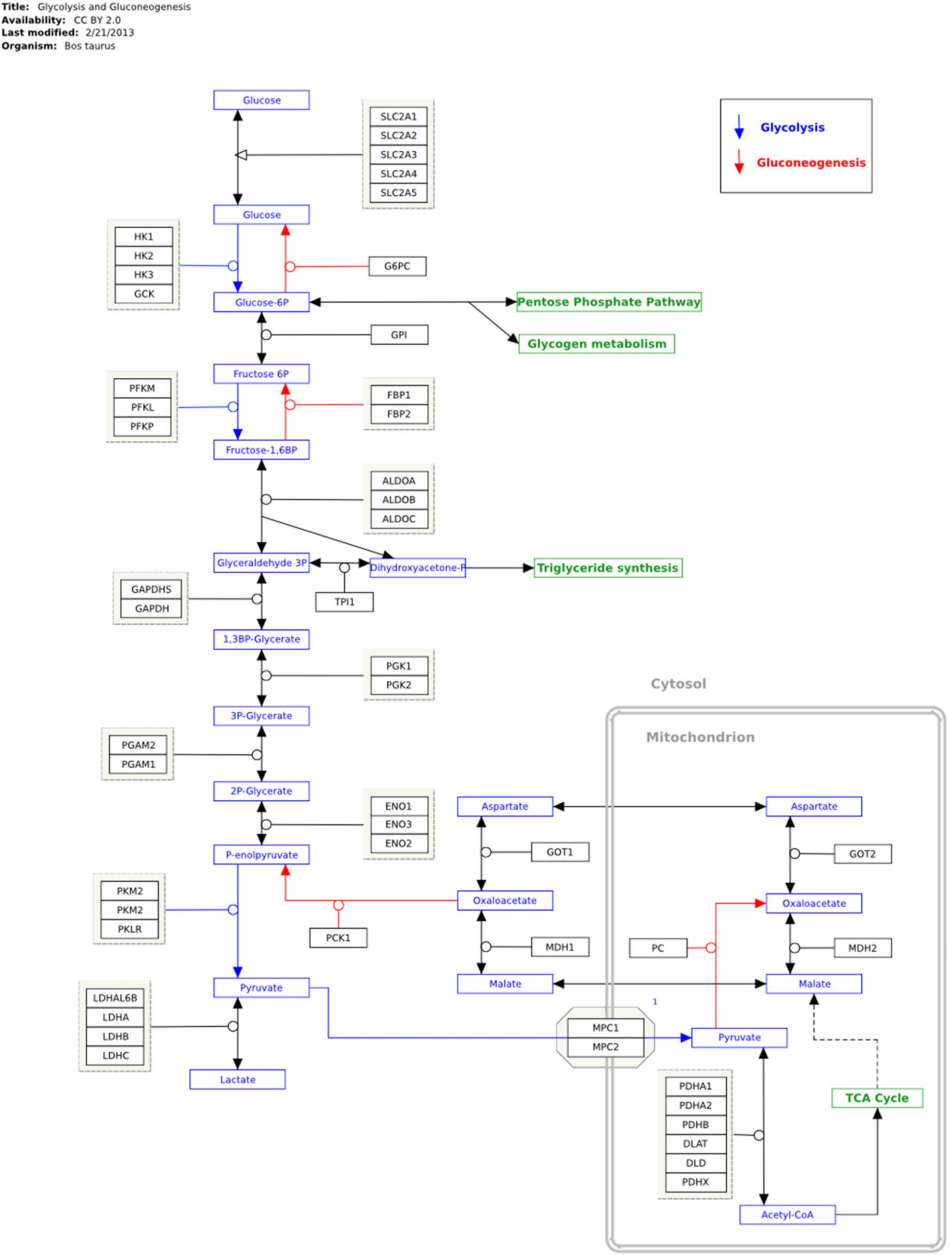
Expression protein profile encoding enzymes in glycolysis and gluconeogenesis pathway. KEGG key: EC 4.1.2.13: Fructose-bisphosphate aldolase (ALDOB); EC 5.3.1.1: Triosephosphate isomerase (TPI); EC 1.2.1.12: Glyceraldehyde-3-phosphate dehydrogenase (GAPDH); EC 5.4.2.4: Phosphoglycerate mutase (PGAM); Alpha-enolase (ENO1); EC 4.2.1.11 Beta-enolase (ENO3); EC 2.7.1.40 Pyruvate Kinase (PKM); EC 1.1.1.27 L-lactate dehydrogenase (LDH).

Additionally, the differential expression indicates similar encoding enzymes in the glycolysis and gluconeogenesis pathways in cattle’s large intestines under different feeding strategies (Fig. 2).

**Figure 3:**
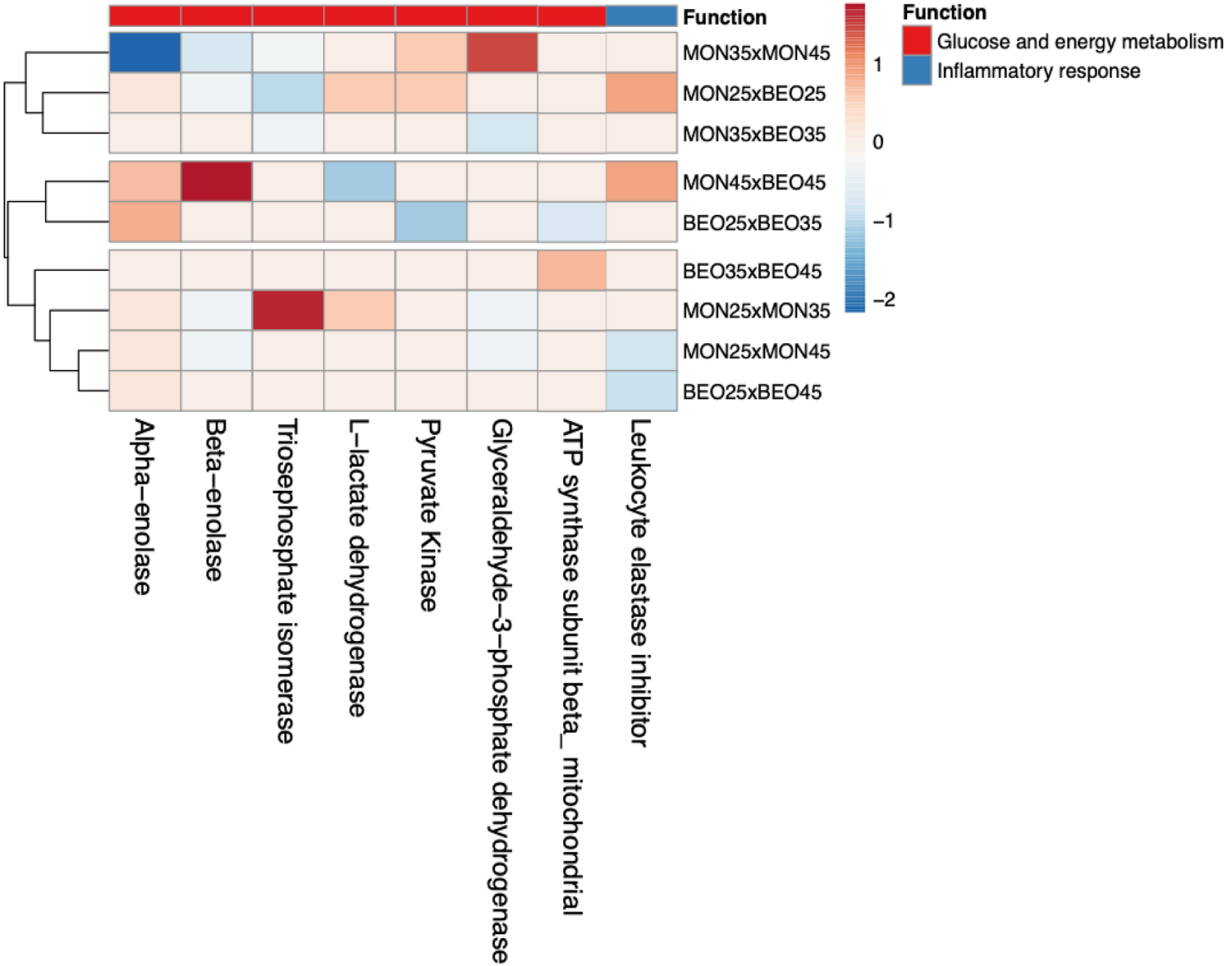
Heatmap of the differentially expressed proteins (ANOVA, *P* $\le$ 0.05) among the diets contending different starch levels and additives. Color-coded matrix showed the correlation coefficient of the *spots* expression values. Each row and column represent one group and protein, respective.

The expression values ($P \le 0.05$) (Table 3) were grouped with hierarchical cluster analysis (Fig. 3) and ordered by homogeneity between the treatments tested. Animals fed with identical levels of starch but subjected to different feed additives showed differentiation in proteins that contribute to energy metabolism.

**Table 3:** Expression values (test t, *P* $\le$ 0.05) in Nellore cattle cecum protein profile fed starch levels (25, 35 and 45%) and additives (monensin and blend essential oil + α-amylase).

Protein	MON × BEOα			MON			BEOα		
	25	35	45	25 × 35	35 × 45	25 × 45	25 × 35	35 × 45	25 × 45
**Glucose and energy metabolism**									
Alpha-enolase	+/∅	NS	1.55	+/∅	− 1.48	+/∅	1.65	NS	+/∅
Beta-enolase	∅/+	NS	1.55	∅/+	− 1.48	∅/+	NS	NS	NS
Triosephosphate isomerase	− 3.55	− 2.55	NS	+/∅	− 2.39	NS	NS	NS	− 1.84
l-lactate dehydrogenase	∅/+	NS	− 1.47	∅/+	NS	NS	NS	NS	NS
l-lactate dehydrogenase B	∅/+	NS	NS	NS	NS	NS	NS	NS	NS
l-lactate dehydrogenase A chain									
Pyruvate kinase	∅/+	NS	NS	NS	∅/+	NS	− 2.54	NS	NS
Fructose-bisphosphate aldolase	NS	NS	− 1.47	NS	NS	NS	NS	NS	NS
Phosphoglycerate mutase									
Glyceraldehyde-3-phosphate dehydrogenase	NS	− 1.49	NS	∅/+	+/∅	∅/+	NS	NS	NS
ATP synthase subunit beta_mitochondrial	NS	NS	NS	NS	NS	NS	∅/+	+/∅	NS
**Inflammatory response**									
Leukocyte elastase inhibitor	+/∅	NS	+/∅	NS	NS	− 1.22	NS	NS	− 1.29

The values are presented in the form log2FC (Fold Change) calculated in relation to the type of additives used, and subsequently the level of starch with the respective additives.

*NS* not significant, +/∅ *spot* present in the first group in relation to the second, ∅/+ *spot* absent in the first group in relation to the second.

The animals fed low-starch diets (25%) associated with BEOα exhibited increased expression of pyruvate kinase (EC 2.7.1.40), beta-enolase (EC 4.2.1.11), triosephosphate isomerase (EC 5.3.1.1), and l-lactate dehydrogenase (EC 1.1.1.27) compared with those treated with monensin; all of these proteins are enzymes catalyzing the synthesis of pyruvate, which is responsible for the degradation of carbohydrates. Animals fed diets with the highest level of starch tested (45%) exhibited greater synthesis of l-lactate dehydrogenase (EC 1.1.1.27), fructose-bisphosphate aldolase (EC 4.1.2.13), and phosphoglycerate mutase (EC 5.4.2.4). Meanwhile, animals fed the intermediate starch level exhibited a higher expression of triosephosphate isomerase (EC 5.3.1.1) and glyceraldehyde-3-phosphate dehydrogenase (EC 1.2.1.12).

## Discussion

When evaluating the tested additives, we found that the use of BEOα with intermediate levels of starch (35%) resulted in greater expression of glycolysis intermediates, thus, this additive may have a greater effect on the post-rumen tract. A companion study (data under review) reported that the optimum level of dietary starch for cattle fed MON was 25%; however, the optimum level of dietary starch for cattle receiving BEO was 35%, commensurate with protein expression synthesis. In summary, feed intake decreased when MON- and BEO-fed cattle were fed more than 25% and 35% starch, respectively, which resulted in decreased average daily gain and proteins linked with carbohydrate degradation. The diets with 45% starch may have caused excessive ruminal fermentation that may have resulted in increased inflammation of the ruminal epithelium, agreeing with the proteins leukocyte elastase inhibitor. The literature reports that increasing levels of starch play an important role in reducing feed intake (observed in a companion study); however, this effect was more evident in cattle that were fed MON as it is a feed additive that depresses intake.

In the protein *spots* of groups 25BEOα and 45BEOα compared with those fed MON, leukocyte elastase inhibitor, a serine protease inhibitor that is essential for the regulation of inflammatory responses and limits the activity of inflammatory caspases, was not expressed^22^ aligning with the results of the above-mentioned authors, who reported reduced ionophore effects in the hindgut. When comparing diets with 25% or 45% starch, regardless of the additive used, leukocyte elastase inhibitor was expressed more, corroborating previous studies demonstrating that inflammatory injuries are caused by the increased use of concentrates in the diet^23,24^. Additionally, we observed reduced expression of proteins that participate in energy metabolism in animals on high-starch diets, which can damage the epithelium of the cecum.

In a similar study, Toseti et al.^9^ observed a reduction in fecal starch using BEOα, suggesting a greater degradation of carbohydrates because the feed enzymes can remain active in the intestine and aid in the digestion of nutrients that escape rumen fermentation^25^. As Thomas et al.^26^ demonstrated, the effect of monensin is more evident in the rumen, mainly in the diversity of microorganisms, but less than 10% results in post-ruminal action, corroborating the hypothesis that ionophore antibiotics have a limited effect on ruminants’ microbiota and intestinal fermentation.

Protein expression differs depending on the dietary starch level ($P \le 0.05$); the cluster analysis shows differentiation in the profile of the identified proteins involved in energy metabolism (Fig. 3) but the effect is greater when contrasting 25% and 35% starch or 35% and 45%, mainly when using monensin as a feed additive. In summary, high concentrations of starch may result in inflammatory responses due to the greater supply of undegradable starch in the rumen, thus decreasing the expression of proteins linked to the glycolytic pathway through tissue damage and inflammation. Higher concentrations of carbohydrates (starch) in the intestine along with the low effects of monensin on the cecum may contribute to a greater accumulation of organic acids. Additionally, the intestinal epithelium is much more vulnerable to pH variation than the rumen^21,27^, corroborating our identification of proteins linked to immune responses. Notably, this was not observed when assessing the full range of starch levels (25% vs. 45%) but the proteins involved in inflammatory responses were expressed more (Fig. 3). We attribute this to the greater increase in dietary carbohydrate, which may have increased epithelial injury (indicative damage)^21,28^ and upregulated inflammatory response, subsequently reducing the expression of proteins associated with energy metabolism.

Fructose-bisphosphate aldolase (ALDOB), an enzyme that converts fructose-1,6-bisphosphate to fructose 6-phosphate, catalyzed by triosephosphate isomerase (TPI), is a precursor of glyceraldehyde-3-phosphate (GA3P), which is acted upon by the enzyme glyceraldehyde-3-phosphate dehydrogenase (GAPDH) during glycolysis. Alpha-enolase (ENO1) and beta-enolase (ENO3) are isoforms of enolase that are involved in Step 4 of glycolytic metabolism. Phosphoglycerate mutase (PGM) is a catalytic enzyme that converts 3-phosphoglycerate to 2-phosphoglycerate and, finally, pyruvate kinase (PKM) synthesizes pyruvate in the last step of glycolysis (UniProt^29^). In ruminants, a high concentration of starch enables the fermentation of carbohydrates in the cecum with lactate production, which increases glucose metabolism in the intestine and leads to the observed expression of the enzyme l-lactate dehydrogenase and its isoforms l-lactate dehydrogenase B and l-lactate dehydrogenase A, which synthesize lactate from pyruvate (UniProt^29^).

The dietary manipulation verified the expression of the leukocyte elastase inhibitor protein, associated with the inflammatory response (Table 2); this plays an essential role in regulating the innate immune response, inflammation, and cellular homeostasis, and mainly acts to protect cell proteases released into the cytoplasm during stress or infection^29^.

## Methods

The experiment was conducted according to the standards issued by the National Council for Animal Experimentation Control (CONCEA) and approved by the Ethics and Use of Animals Committee of the São Paulo State University (UNESP, Botucatu-SP), under protocol no 0107/2019 and in compliance with ARRIVE (animal research: reporting of in vivo experiments) guidelines^30^.

### Animals, facilities, feeding and animal care

The animal experiment was conducted at the feedlot facilities of the Innovation and Applied Science Center of DSM Nutritional Products (I & AS Beef Center; Rio Brilhante, Mato Grosso do Sul, Brazil). Nellore bulls (n = 210) (*Bos taurus indicus*) from the grazing system with an average body weight of ± 380 kg were used. The animals were randomly allocated to pens (7 animals/pen) with 12 m^2^/animal and collective troughs (50 cm linear/animal). The program for receiving the animals consisted of weighing, deworming, and vaccinating according to the annual prophylactic calendar. The animals underwent a pre-experimental adaptation period of 10 days to standardize their rumen population and allow them to adapt to the facilities and management. The diets were formulated with the LRNS system (large ruminant nutrition system^31^), level 2, meeting the nutritional requirements for daily weight gain between 1.5 and 1.7 kg/animal. Animals were fed for 92 days and diets were offered ad libitum twice daily at 8 a. m. and 3 p.m.

### Experimental design

A factorial 3 × 2 arrangement was used, with starch level (25%, 35%, or 45%) and additives (monensin or the essential oil blend CRINA^®^ with the exogenous α-amylase Rumistar^®^) as the factor. The sodium monensin (MON; Rumensin, Elanco Animal Health, Indianapolis, IN) used was included in the diet at a dose of 26 mg/kg of dry matter. The blend of functional oils (CRINA RUMINANTS^®^; DSM Nutritional Products, Basel, Switzerland) containing thymol, eugenol, limonene, and vanillin^32^ and the exogenous enzyme α-amylase (RONOZYME RUMISTAR™; DSM Nutritional Products, Basel, Switzerland), referred to as BEOα were added to the diet at a dose of 90 mg/kg of dry matter and 560 mg/kg of dry matter, respectively. The pens were distributed in a randomized block design, totaling 6 treatments with 5 repetitions or 30 experimental units overall. The treatments were distributed within the blocks as follows: T1 (25MON), T2 (25BEOα), T3 (35MON), T4 (35BEOα), T5 (45MON), and T6 (45BEOα). According to the statistical model:

$$Y_{ijk} = \mu + B_{k} + C_{i} + A_{J} + \left( {C \times A} \right)_{ij} + \varepsilon_{ijk} ,$$

where $Yijk$ is the dependent variable; $\mu$ is the overall mean; $Bk$ is the block effect; $Ci$ is concentrate; $AJ$ is additive; $(C \times A)ij$ is the interaction between concentrate and additive effects; and $\varepsilon ijk$ is the residual error.

### Diets and their chemical composition

The experimental diets were composed of natural bagasse sugarcane, ground corn, soybean hulls, cottonseed, soybeans, core minerals and vitamins, urea, and additives. The transition to the finishing diet was managed as follows: for 14 days, two diets with 65% and 75% concentrate were provided for 7 days each. From the 15th day of the experiment until slaughter, a finishing diet containing 85% concentrate was provided (Table 4).

**Table 4:** Aining increasing starch levels (25, 35, and 45%) and additives (monensin, blend of essential oil + exogenous α-amylase) in diets for Nellore cattle feedlot.

Starch level (%)	Diets								
	25			35			45		
	Adap. 1	Adap. 2	Finishing	Adap. 1	Adap. 2	Finishing	Adap. 1	Adap. 2	Finishing
**Ingredients (g/kg)**									
Sugarcane bagasse	350	250	150	350	250	150	350	250	150
Corn grain grind	300	330	360	300	400	500	300	470	640
Soybean meal	90	55	20	90	65	40	90	75	60
Whole cottonseed	60	80	100	60	80	100	60	80	100
Soybean hulls	150	235	320	150	155	160	150	75	0
Mineral and vitamin supplement	50	50	50	50	50	50	50	50	50
**Nutrient content (dry matter, g/kg)**									
CP	146	147	146	146	147	146	146	145	145
TDN	660	680	690	660	690	730	660	720	770
DPI	510	510	500	510	510	520	510	520	530
NDF	437	424	412	437	382	330	437	316	252
peNFD^7^	360	300	250	360	290	230	360	280	220
Ca	7.7	7.5	7.3	7.7	7.5	7.3	7.7	7.6	7.5
P	3.1	2.8	2.5	3.1	3.1	3.1	3.1	3.6	3.7
Starch	209.5	230.8	254.6	209.5	284.0	355.0	209.5	372.8	458.0
NE Mcal/kg DM	2.4	2.4	2.4	2.4	2.5	2.6	2.4	2.6	2.7

*Adap 1* adaptation 1, 0–7 days, *Adap 2* adaptation 2, 7–14 days, 14–92 days, *CP* Crude protein, *TDN* total digestible nutrients, *DPI* digestible protein intake, *NDF* Neutral detergent fiber, *peNFD* physically effective neutral detergent fiber, *Ca* calcium, *P* phosphor, *NE* net energy.

Dietary energy content was calculated according to the LRNS system^31^ and Total digestible nutrients (TDN) were determined by the equation: TDN = digestible CP + (digestible EE × 2.25) + digestible NDF + digestible non-structural carbohydrate (NSC). Crude protein was determined by assessing the nitrogen content of the samples with the Kjeldahl method^33^. The NDF concentration was assessed with the methodology described by Van Soest et al.^34^ and corrected for CP and ashes. Starch was determined by the equation: NSC = 100 − CP − EE − NDF − ash, where ash content was determined by incinerating samples at 550 °C for 2 h in a muffle furnace^35^. Physically effective neutral detergent fiber (peNFD) was determined according to Kononoff et al.’s methods^36^. Samples of diets were collected to determine particle-size distribution by sieving with the Penn State particle-size separator and reported on an as-fed basis.

### Proteomics sample collection and preparation

The animals were transported to a commercial slaughterhouse where they were stunned by brain concussions with a captive dart gun. After bleeding hide removal and evisceration, cecum samples about 4 cm square were collected and washed with phosphate-buffered saline (PBS), transferred to 15 mL polypropylene bottles, and placed in liquid nitrogen (− 196 °C) for later protein extraction. Each pen was considered an experimental unit, so a pool of samples was made by homogenizing cecal tissue from animals given the same treatment; three animals per experimental unit (N = 5) were used, i.e., 15 animals/group or 90 animals total (15 animals from each of six groups).

### Extraction, precipitation and quantification of proteins

To extract the protein fraction, the tissue was macerated with a mortar and pestle in the presence of liquid nitrogen. The extracting solution was added at a rate of 1 mL ultrapure water per 1 g tissue and then the samples were homogenized with an OMMI-BEAD RUPTOR4 cell disruptor (Kennesaw, Georgia, United States) over three 30-s cycles. The samples were then separated into protein extracts and the supernatant was collected after refrigerated centrifugation (− 4 °C) with a UNIVERSAL 320R HETTICH (Tuttlingen, Baden-Württemberg, Germany). The proteins were precipitated in 80% (v/v) acetone (J.T. Baker, Phillipsburg, New Jersey, United States), using 300 μL of supernatant and 600 μL of 80% acetone. The samples were stored at 2 °C for 1.5 h and then centrifuged at 14,000 rpm for 30 min; the supernatant was discarded and the protein pellet was solubilized in 1 mL of 0.50 mol/L NaOH (Merck, Darmstadt, Germany). The protein concentrations were determined by the Biuret method^37^, using an analytical curve with a concentration range of 0–100 g/L of standard bovine albumin solution (Acros Organics, NJ, United States) at 100 g/L.

### Electrophoretic separations of protein fractions using 2D-PAGE

For isoelectric focusing, about 375 µg of proteins from each group were applied to individual strips; the sample was resolubilized with a solution containing 7 mol/L urea, 2 mol/L thiourea, 2% CHAPS (m/v) (GE Healthcare, Uppsala, Sweden), ampholytes at a pH of 3 to 10 at 0.5% (v/v) (GE Healthcare, Uppsala, Sweden), and 0.002% bromophenol blue (GE Healthcare, Uppsala, Sweden), in addition to 2.8 mg of dithiothreitol (USB, Cleveland, Ohio, United States). Approximately 900 µL of mineral oil was added at room temperature for 12 h to rehydrate the strips and prevent evaporation and urea crystals. After this period, the strips were added to the EttanTMIPGphorTM3 isoelectric focusing system (IEF) (GE Healthcare, Uppsala, Sweden). The electrical voltage used was established by the protocol described by Braga et al.^38^. At the end of the focusing period, the strip was balanced in two 15-min stages. First, 10 mL of a solution containing 6 mol/L urea, 2% SDS (w/v), 30% glycerol (v/v), 50 mmol/L Tris–HCl (pH 8.8), 0.002% bromophenol blue (w/v), and 2% DTT (w/v) was used to keep the proteins in their reduced forms^38,37^. In the second stage, a solution in which DTT was replaced with 2.5% (w/v) iodoacetamide was used to alkylate the thiol groups of the proteins and prevent possible reoxidation. After strip balancing, the second portion of the electrophoretic process (SDS-PAGE) occurred.

The strip was applied to a 12.5% (w/v) polyacrylamide gel previously prepared on a glass plate (180 × 160 × 1.5 mm). The gel was placed next to the strip with a piece of filter paper containing 6 µL of a molecular mass standard (GE Healthcare, Uppsala, Sweden), with proteins of different molecular masses (β-phosphorylase [97.0 kDa], albumin [66.0 kDa], ovalbumin [45.0 kDa], carbonic anhydrase [30.0 kDa], trypsin inhibitor [20.1 kDa], and α-lactalbumin [14.4 kDa]). The strip and filter paper were sealed with 0.5% agarose solution (w/v) to ensure contact with the polyacrylamide gel. The run program was then applied at 100 V for 30 min and a further 250 V for 2 h. After the run period, the gels were immersed in a fixative solution containing10% acetic acid (v/v) and 40% ethanol (v/v) for 30 min. Then, the proteins were revealed with colloidal Coomassie G-250 (USB, Cleveland, Ohio, United States) for 72 h and removed by washing with ultrapure water^38–41^.

The gels obtained (Supplemental Fig. S1) were scanned and their images analyzed with the image processing program ImageMaster 2D Platinum 7.0 (GeneBio, Geneva, Switzerland; www.gelifescience.com), which allows the estimation of the isoelectric points and molecular masses of the separated proteins and calculation of the number of *spots* obtained via gel electrophoresis. Three replicates of each gel were used to evaluate the reproducibility of each protein *spot* obtained in the replicates of the gels by overlaying the image from one gel over the other in the image treatment program^39–42^.

### Protein identification by mass spectrometry (LC–MS/MS)

The differentially expressed *spots* were characterized via mass spectrometry after the identification was standardized according to the highest protein score, pI, and molecular mass (MM) closest to the theoretical and experimental results. Among the proteins identified, 12 were classified as functional for this study as they are related to energy metabolism and inflammatory responses.

The protein *spots* were characterized with LC–MS/MS after being subjected to tryptic digestion and peptide elution according to the methodology Shevchenko et al.^43^ described. The aliquots of the solutions containing the eluted peptides were analyzed to obtain the mass spectra with the nanoAcquity UPLC system coupled to the Xevo G2 QTof mass spectrometer (Waters, Milford, MA, United States). Proteins were identified by searching in the UniProt database (www.uniprot.org) within the *Bos taurus* species. Proteins were considered depending on their theoretical and experimental isoelectric points, molecular masses, and scores (> 60). After identifying FASTA sequences in the proteins, their sequences were analyzed with OMICSBOX software (BLAST2GO)^44^ and they were categorized by their molecular function, biological processes, and biochemical activities with gene ontology (GO).

### Proteomic statistical analysis

The starch level and additive were the fixed effects analyzed in a factorial design; thus, the groups were compared through contrasts to verify differentially expressed protein *spots*. Only proteins with significantly altered levels were selected for identification by MS. The images were analyzed with ImageMaster Platinum software version 7.0, which establishes correlations (matching) between groups. For this correlation, three gel replicates were compared for volume, distribution, relative intensity, isoelectric point, and molecular mass in an analysis of variance (ANOVA) with a *t-*test to determine the significance of differentially expressed protein *spots*.

Following the average mode of background subtraction, individual *spot* intensity volume was normalized with total intensity volume (the summation of the intensity volumes obtained from all *spots* in the same 2-DE gel). The normalized intensity volume values of individual protein *spots* were then used to determine differential protein expression among experimental groups. A heatmap showed the correlation coefficient of the *spot* expression values and, after checking the differentially expressed *spots* (*t*-test, *P* < 0.05), the log2 FC values were used for hierarchical cluster analysis.

### Pathways enrichment analysis

The same KEGG-IDs were used to analyze metabolic pathways using the Kyoto Encyclopedia of Genes and Genomes function (KEGG pathways)^45–47^ and Reactome pathway enrichment analysis yielded similar results about the specific pathways affected, allowing the expressions of proteins encoding enzymes found in the database to be mapped.

## Conclusions

In verifying the differential expression of the cecal proteome in cattle, our results show that the blend of essential oils associated with α-amylase incorporated as a feed additive for beef cattle increased the expression of enzymes at dietary starch levels of 25%, 35%, and 45% compared with monensin. The higher expression of proteins related to carbohydrate degradation that participate in glycolysis and gluconeogenesis depended on increased feed intake and reduced protein synthesis expression. The optimum starch level was 35% for both feed additives; higher concentrations of starch (45%) increased the expression of inflammatory responses and reduced the expression of proteins involved in energy metabolism, probably due to damage to the cecum epithelium.

## Supplementary Information

Supplementary Information.

## Data Availability

The datasets used can be made available by the corresponding author on reasonable request.
