# Supplementary material for: Feedlot diets containing different starch levels and additives change the cecal proteome involved in cattle’s energy metabolism and inflammatory response
Source: Sci Rep. 2022 Apr 5;12:5691. doi: 10.1038/s41598-022-09715-7 (PMC8983758; doi:10.1038/s41598-022-09715-7)
Supplement: Supplementary file 1 — Supplementary Information. [file 41598_2022_9715_MOESM1_ESM.docx]

**Supplemental Figure 1*.*** Polyacrylamide gel electrophoresis images, with 12.5% (m/v) obtained by 2D-PAGE electrophoresis;13 cm polyacrylamide gel strips containing immobilized ampholytes with pH gradients at 3 to10; T1: 25 % starch and Monensin; T2: 25 % starch and Blend Essential Oil + α-Amylase; T3: 35 % starch and Monensin; T4: 35 % starch and Blend Essential Oil + α-Amylase; T5: 45 % starch and Monensin; T6: 45 % starch and Blend Essential Oil + α-Amylase.


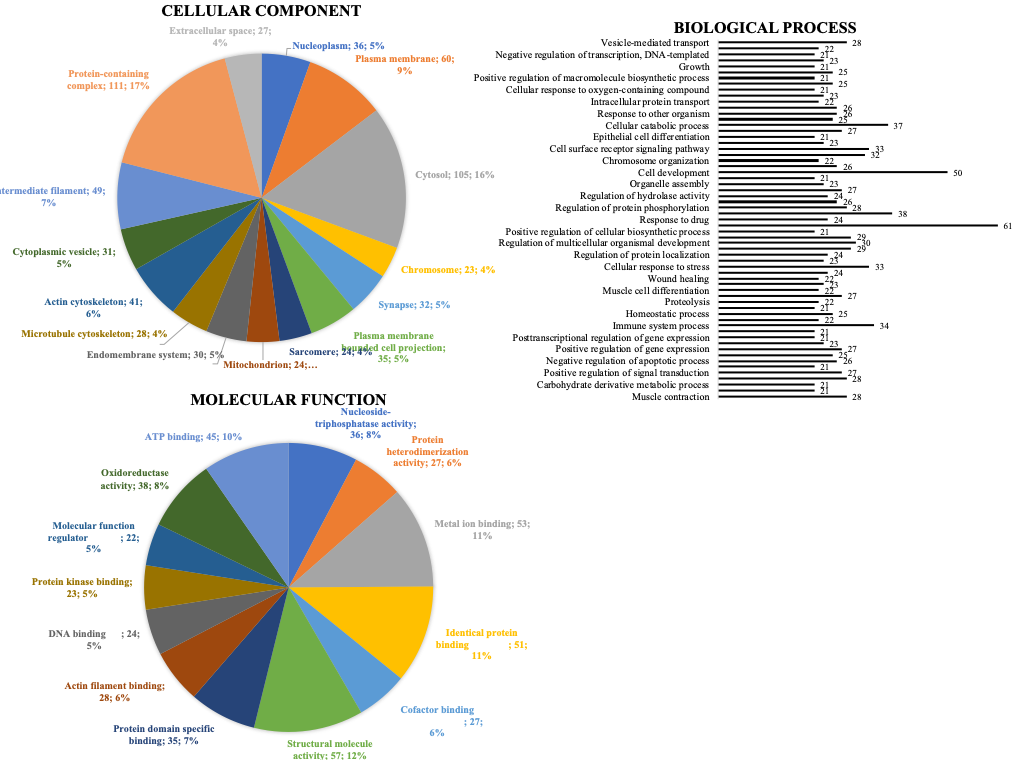


**Supplemental Figure 2.** Classification of the proteins sequences found in beef cattle cecum proteome using OMICSBOX software analysis (Blast2GO).

**Supplemental Table 1.** Values of Reactome Statistical analysis.

| **Pathway** | **Raw P.value** | **FDR Adjusted P.Value** |
| --- | --- | --- |
| Pyruvate Metabolism | 3.2E-2 | 1.58E-1 |
| Gluconeogenesis | 3.51E-2 | 1.58E-1 |
| Pyruvate Metabolism and Citric Acid (TCA) Cycle | 5.62E-2 | 1.58E-1 |
| Glycolysis | 7.88E-2 | 1.58E-1 |
| Glucose Metabolism | 9.81E-2 | 1.7E-1 |
| The Citric acid (TCA) cycle and respiratory electron transport | 1.7E-1 | 1.7E-1 |
| Metabolism Carbohydrate | 2.73E-1 | 2.73E-1 |
| Neutrophil degranulation | 4.02E-1 | 4.02E-1 |
| Metabolism | 6.88E-1 | 6.88E-1 |
| Innate Immune System | 7.32E-1 | 7.32E-1 |
